# An integrated Java tool for generating amino acid sequence alignments with mapped secondary structure elements

**DOI:** 10.1007/s13205-014-0222-0

**Published:** 2014-05-20

**Authors:** Conan K. Wang, Andreas Hofmann

**Affiliations:** 1 Institute for Molecular Biosciences, University of Queensland, St Lucia, QLD Australia; 2 Structural Chemistry Program, Eskitis Institute, Griffith University, Brisbane, QLD Australia; 3 Faculty of Veterinary Science, The University of Melbourne, Parkville, VIC Australia

**Keywords:** Modelling, Structure analysis, Structure-based amino acid sequence alignments

## Abstract

The mapping of secondary structure elements onto amino acid sequences enhances the quality of alignments frequently used in phylogenetic, genomic and transcriptomic studies, as well as in molecular modelling. Here, we report recent updates to the Java application SBAL, an integrated tool to generate, edit, visualise and analyse secondary structure-based sequence alignments. The main goal of the software is to streamline the work flow in generation of structure-based alignments, and we have thus implemented the option to import and visualise sequence and structure information directly from any PDB file. The new feature is achieved by a Java application named ASSP which follows the original framework of the well-established dictionary of protein secondary structure by Kabsch and Sander. ASSP is also available as a stand-alone application. Other major additions to SBAL include the calculation of distance matrices, peptide properties, as well as detailed on-line tutorials for typical applications.

**Electronic supplementary material:**

The online version of this article (doi:10.1007/s13205-014-0222-0) contains supplementary material, which is available to authorized users.

## Introduction

The rate of completion of new genome sequencing projects has been rapidly increasing in the recent past, thus providing large amounts of information on new proteins. To characterise and classify the immense number of new proteins from genome and transcriptome projects, automated assignment methods are used that, in the majority of cases, correctly annotate a new protein sequence to a known homologous protein fold (Cantacessi et al. 2010, 2014). Assembled nucleotide sequences from genomic and transcriptomic studies are usually conceptually translated into predicted proteins using algorithms that identify protein-coding regions. The predicted peptide sequences are then analysed for protein identity, for example with the software InterProScan (Hunter et al. 2012), by comparison of sequences with data available in public databases, to infer known protein domains. However, this first-pass annotation does not reveal any specific molecular features of the annotated proteins, such as conservation of active sites, variations of the conserved fold (Osman et al. 2012), or novel structural elements (Cantacessi et al. 2013).

To gain more detailed insights at the molecular level, in the absence of an experimental three-dimensional structure, comparative modelling can be employed. In many cases, this results in generation of three-dimensional atomic models of the target protein. Frequently, however, essential details can be gleaned from appropriate amino acid sequence alignments. In this context, informed sequence alignments are essential for constructing motifs, profiles and atomic modelling instructions (Eidhammer et al. 2000; Hubbard and Blundell 1987; Marchler-Bauer et al. 2002; Sauder et al. 2000). Often, amino acid sequence identities between two distantly related proteins are rather low when comparing, for example, parasite with vertebrate proteins (A Jex & RB Gasser, pers. commun.), although there are a few exceptions (Hewitson et al. 2009). In such situations, it is difficult to obtain meaningful alignments based on amino acid sequence similarity alone (Marchler-Bauer et al. 2002; Sauder et al. 2000). Since the main criterion for structural homology of two proteins is that they adopt the same fold, structure-based amino acid sequence alignments have been used as the gold standard for sequence alignment evaluation (Hubbard and Blundell 1987; Russell and Barton 1994).

We have previously developed the Java application SBAL (Wang et al. 2012) to fill an apparent gap in the seamless transition from secondary structure-based sequence alignments to the visualisation of the results. The main emphasis in the development of this software was the ease-of-use and the integration of transitional steps such as reformatting and editing, integrated with aids for visualisation and analysis. Among the variety of input formats for the original SBAL software, sequence and secondary structure information could be read directly from experimental three-dimensional structures in protein data bank (PDB) format. This could be achieved either by reading information from the PDB header section (‘HELIX’ and ‘SHEET’ records) or externally processing the PDB file with the established software DSSP (Kabsch and Sander 1983), which assigns secondary structure information to each residue based on hydrogen bonding patterns. In many practical environments, however, we found that not all structures in PDB format contain ‘HELIX’/‘SHEET’ records (e.g. if they are not obtained from the PDB, but become accessible through ongoing work), and that in such cases the dependence on external non-Java software DSSP presents an inconvenient break in the work flow. Furthermore, this new tool also allows direct visualisation of the amino acid sequence of a three-dimensional structure in PDB format with automatically mapped secondary structure elements.

Here, we report on an update of the SBAL software which includes improved parsers to better handle the variety of input file formats, as well as further processing and analysis tools. We also implemented the DSSP algorithm into Java and introduce the new application analysis of secondary structure of proteins (ASSP). ASSP has been embedded with the new version of SBAL, thus eliminating the need for running DSSP as an external programme in a separate step prior to executing SBAL, thus creating a streamlined and user-friendly platform for sequence-structure analyses. ASSP is also available as a stand-alone application to analyse secondary structure of three-dimensional models in PDB file format and can be integrated into other Java applications.

## Programme implementation and methods

### ASSP working concept

The Dictionary of Protein Secondary Structure (DSSP) was designed by Wolfgang Kabsch and Christian Sander to standardise secondary structure assignment (Kabsch and Sander 1983). DSSP has since become a database of secondary structure assignments and other data for all protein entries in the PDB (Joosten et al. 2011). The DSSP software is available as a web service or stand-alone programme, written in C++ (http://www.cmbi.ru.nl/dssp.html). Despite its popularity and the eminent importance of the concept for structural biology, the algorithm appears not to have been ported to other programming languages.

In the context of our Java programme collection for structural biology and biophysical chemistry (PCSB) (Hofmann and Wlodawer 2002), and specifically for the application SBAL (Wang et al. 2012), a tool for structure-based sequence alignments, we have implemented the DSSP algorithm as described in the original report by Kabsch and Sander (1983) in Java. To avoid naming confusion with the original DSSP, the Java application is called ASSP.

ASSP works in a similar fashion as DSSP, and its Java API also allows for integration with other Java software. As a stand-alone application, the user can read a three-dimensional protein structure in PDB format and the software will analyse all geometric and secondary structure (H-bonding) parameters. Parsing of the PDB file is accomplished by the PDB file class of PCSB, and includes a check for hydrogen atoms being present. If so, the provided hydrogen atoms are kept and used for analysis. If no hydrogen atoms are present in the PDB file, the amide hydrogen atoms will be modelled.

### ASSP output

Results are reported either to the terminal or can be saved into an ASCII file. The user can choose to have the results output in DSSP format (to allow for comparison with DSSP, we have used the same format as that delivered by the DSSP web service at http://www.cmbi.ru.nl/hsspsoap/) or in ASSP format. We believe the ASSP output format to be more convenient for visual inspection and manual analysis. Apart from a more generous use of spaces between individual parameters, ASSP reports all residues by their original name as listed in the PDB file. For comparison, DSSP addresses residues by a sequential index.

### SBAL

SBAL is a tool to visualise, generate and edit secondary structure-based sequence alignments (Wang et al. 2012). Users can choose to either generate secondary structure-based alignments using SBAL or import sequences with or without secondary structure information from a variety of established input formats, including FASTA, MSF, ClustalW (Larkin et al. 2007), PSIPRED (Bryson et al. 2005), DSSP (Kabsch and Sander 1983) and PDB. When importing information from three-dimensional structures, the secondary structure assignments so far relied on the ‘HELIX’ and ‘SHEET’ records in PDB files, or else the user previously needed to pre-process the structure in PDB format with the software DSSP.

**Fig. 1:**
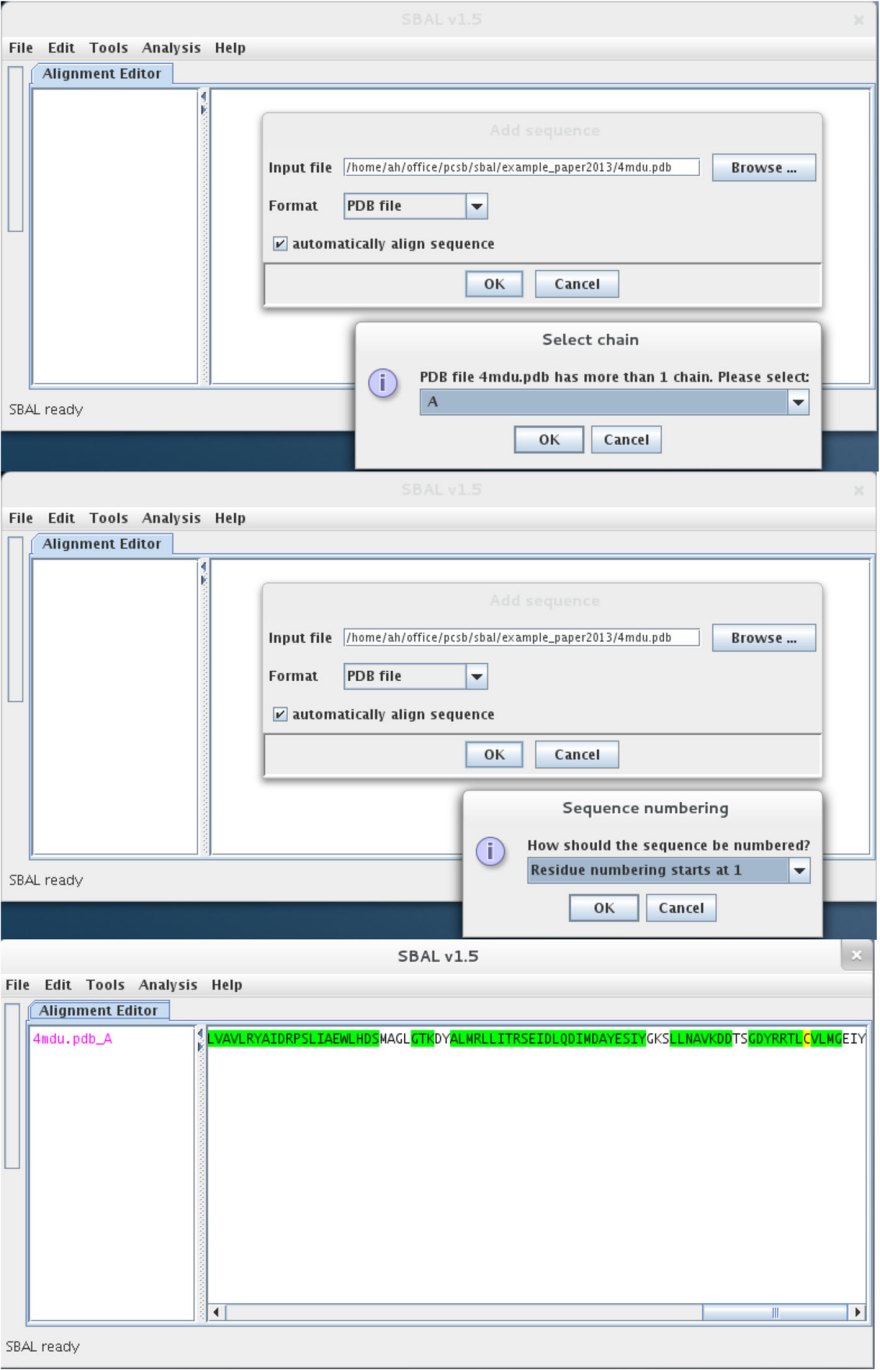
Screen shots of importing PDB file 4mdu (Leow et al. 2014) into SBAL. *Top panel* SBAL recognises the presence of different chains in the file and prompts the user to choose which chain should be imported. *Middle panel* If the starting residue number differs from ‘1’, the user can choose to retain the residue numbering as in the PDB file or re-number from 1. *Bottom panel* Since the PDB file did not have any ‘HELIX’/‘SHEET’ records, SBAL automatically assigns secondary structure using the embedded version of ASSP. Helical structure is indicated in *green*, β-strands are coloured *red*

ASSP has been embedded into the SBAL application and, when loading a PDB file, the user is prompted with a choice of using the secondary structure information from the ‘HELIX’ and ‘SHEET’ records in the PDB file, or analysing the structure with the built-in ASSP module. Although the import of information from DSSP is still possible, use of the built-in ASSP module provides an extra level of user-friendliness (see Fig. 1).

### Availability

Both programmes make use of and extend Java classes previously developed in our laboratory (Hofmann and Wlodawer 2002; Wang et al. 2012). They are available as stand-alone compiled Java applications from the project home page at http://www.structuralchemistry.org/pcsb/. The ASSP API includes methods that enable interfacing with other Java applications and may thus also be useful to developers. The applications and manuals are freely available to academic users. For download, users will be asked for their name, institution and email address. The source code is available from the authors upon request.

## Results

### Benchmarking of ASSP

**Fig. 2:**
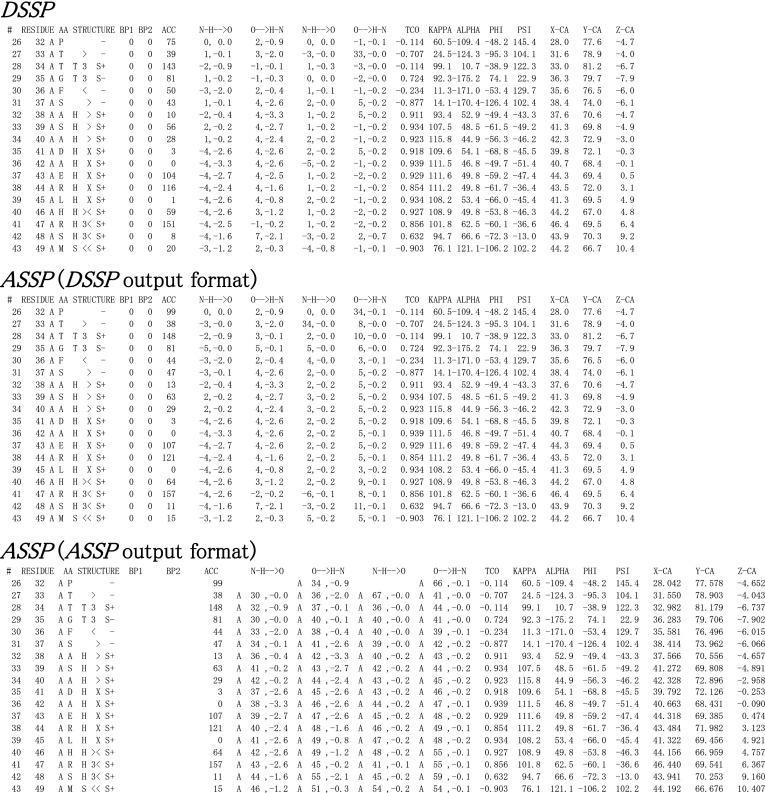
Excerpt from the secondary structure analysis of the same PDB file as in Fig. 1 with DSSP (*top section*), and ASSP (*middle and bottom sections*). The type of information reported by ASSP is the same as that of DSSP. The middle section shows a report generated by ASSP mimicking the DSSP output format with relative referencing of residues. For user convenience, ASSP can also produce reports where residues are referenced with their absolute names, i.e. chain identifier and residue number as in the PDB file (*bottom section*)

To evaluate the secondary structure assignment implementation in ASSP, a dataset of crystal structure files was initially selected using the PISCES server (Wang and Dunbrack 2005), using three criteria: (i) sequence identity below 20 %, (ii) resolution better than 1.6 Å, (iii) R-factor <0.25. This dataset included 2,118 PDB files, from which 100 PDB files (the last 100 of a PDB code-sorted list) were selected as the benchmark set, which comprised over 28,000 residues. The mathematically derived parameters such as hydrogen bonds, angles, etc. reported by ASSP are in excellent agreement with those reported by DSSP. To evaluate the agreement of the secondary structure summary assignment of ASSP with that of DSSP, an in-house script was used to compare the output files of both programmes (see Table 1; example output in Fig. 2). According to DSSP, the benchmark set contains 5,626 residues with no secondary structure assignment, 321 residues in an isolated β-bridge (B), 5,730 residues in an extended strand (E), 1,305 residues in a 3_10_-helix (G), 9,822 residues in an α-helix (H), 24 residues in a π-helix (I), 2,258 residues in a bend (S), and 2,988 residues in a H-bonded turn (T). ASSP was able to achieve between 85 and 100 % agreement for all cases except for assignments of residues in an isolated β-bridge. Discrepancies in the individual assignments arise when the criteria for more than one type of secondary structure assignment are met and a particular type needs to be assigned based on an hierarchy. This is also true for assignment of the 3_10_-helical type (G), where ASSP assigns an H-bonded turn (T) to a fraction of ~15 %, since it only allows the assignment of H, G or I for more than three consecutive residues with those types.

**Table 1:** Distribution of assigned secondary structure types (‘summary’) in the benchmarking dataset analysed with DSSP and ASSP

DSSP assignment	ASSP assignment							
	None	B	E	G	H	I	S	T
None	5,264	150	179	5	23	0	0	5
B	76	156	28	0	2	0	48	11
E	245	49	5,342	2	0	3	73	16
G	24	0	0	909	9	2	160	201
H	3	0	0	547	9,016	104	78	74
I	0	0	0	0	0	24	0	0
S	0	0	16	3	4	0	2,235	0
T	15	5	0	244	81	38	56	2,549

### Mapping of secondary structure elements

When mapping secondary structure elements onto an amino acid sequence, SBAL uses three simplified structure types: helical, extended and unstructured. If the information is extracted from fully annotated PDB files, residue ranges listed as ‘HELIX’ are mapped as ‘helical’, residue ranges annotated with ‘SHEET’ are mapped as ‘extended’, and all other residues are mapped as ‘unstructured’. Accordingly, when using either ASSP or DSSP analysis of three-dimensional structures, the three helical secondary structure types (H, I, G) are combined and mapped as ‘helical’, the extended strand (E) is mapped as ‘extended’, and all other types are reported as ‘unstructured’. From the benchmarking exercise, we find an agreement of ASSP and DSSP results of around 90 % or better (see Table 2).

**Table 2:** Comparison of simplified ASSP and DSSP secondary structure assignment results obtained with the benchmarking dataset

Sec. structure	DSSP	ASSP	Agreement
Unstructured	11,193	10,204	0.912
Helical	11,151	9,949	0.892
Extended	5,730	5,342	0.932

### New features of SBAL

With more than 600 downloads since its publication in 2012, SBAL is one of the most popular Java applications of our programme collection. Based on feedback from users, several improvements have been made to address minor glitches, improve parsing of various input file formats, as well as enhance the user experience.

We have embedded an automated secondary structure analysis tool (ASSP) into SBAL that is automatically deployed when a PDB file without ‘HELIX’/‘SHEET’ records is loaded (see Fig. 1); otherwise, the user is prompted to choose which source of secondary structure information to use. Importantly, all three types of helical secondary structure, 3_10_-helix, α-helix and π-helix, are combined into helical structure at this step.

In the ‘Tools’ section, the calculation of a distance matrix for the current alignment has been added. Pairwise distances for all sequences in the alignment can be calculated based on either the amino acid identity or *p* distances. For the latter, the user has the option to choose *p* distances as used in the EMBOSS programme distmat (Rice et al. 2000), or the programme MEGA (Kumar et al. 2001). The distance matrix is displayed in a separate window and can readily be exported to spreadsheet programmes by a copy–paste operation.

Another addition to the ‘Tools’ section is the option to calculate peptide properties for the currently active sequence in the alignment by just one mouse click. The physical peptide properties calculated include number of amino acids, molecular mass, extinction coefficient at a wavelength of 280 nm, isoelectric point and charge at pH 7 (Hofmann and Wlodawer 2002).

Among the convenience features added are the option to add an annotation to an alignment, a moveable barrier between the ID panel and the sequence panel (to accommodate long sequence titles), and more extensive preference settings that allow changing various colour settings and the number of amino acid residues within one line of the HTML output format. On the project web site (http://www.structuralchemistry.org/pcsb/), a site dedicated to SBAL includes examples of various frequent scenarios with step-by-step instructions.

## Conclusions

Amino acid sequence alignments are core to structural biology and alignments with mapped secondary structure elements are essential to inferring structural homology between proteins. SBAL has proven to be a popular integrated Java tool for generation, visualisation and analysis of structure-based sequence alignments based on the download statistics. With development and implementation of a Java tool that conducts geometric/hydrogen bond analysis of three-dimensional protein structures to automatically derive secondary structure assignments, the work flow in generation of structure-based sequence alignments has been significantly simplified, as PDB files can now be directly accessed for sequence alignment purposes, without the need for pre-processing.

The stand-alone ASSP analysis tool as Java application closes a gap in the currently available Java libraries for structural biology and will be useful in any computational structural biology context. The API will further assist in development of future Java classes for structural biology. The current version of ASSP has several built-in methods aiming particularly at modelling applications (e.g. output of information directly usable in structure-based amino acid sequence alignments), and we plan to implement further model analysis tools in the future.

## Electronic supplementary material

Below is the link to the electronic supplementary material.

ASSP and DSSP results obtained with the benchmarking dataset

Supplementary material 1 (ZIP 15968 kb)
