# Supplementary material for: An integrated Java tool for generating amino acid sequence alignments with mapped secondary structure elements
Source: 3 Biotech. 2014 May 20;5(1):87–92. doi: 10.1007/s13205-014-0222-0 (PMC4327748; doi:10.1007/s13205-014-0222-0)
Supplement: Supplementary file 1 — Supplementary material 1 (ZIP 15968 kb) [file 13205_2014_222_MOESM1_ESM.zip › 3biotech/assp_v0.9_manual.pdf]

ASSP v0.9

Table of Contents

1. SUMMARY.....1
2. BACKGROUND.....1
3. DESCRIPTION OF WORKING CONCEPT.....2
3.1 Output formats.....2
3.2 Comparison of ASSP and DSSP results.....2
4. OPERATION.....3
4.1 From the terminal 3
4.2 Examples.....3
5. EXPLANATION OF OUTPUT FORMATS.....4
5.1 ASSP format.....4
5.2 DSSP format.....5
5.3 Geometric parameters.....6
6. REFERENCES.....7
7. APPENDIX: COPYRIGHT, LICENCE AND DISCLAIMER.....8

1. Summary

ASSP is a Java application for analysis of secondary structure of three-dimensional protein models. It is based on the algorithm used in DSSP (Kabsch & Sander, 1983). A user-supplied structure in form of a PDB file will be analysed for H-bonding and geometric parameters to deliver a secondary structure assignment.
ASSP is part of the Program Collection for Structural Biology and Biophysical Chemistry (PCSB) (Hofmann & Wlodawer, 2002). When using ASSP, please cite Java applications for secondary structure analysis of proteins .

2. Background

The Dictionary of Protein Secondary Structure (DSSP) was designed by Wolfgang Kabsch and Christian Sander to standardise secondary structure assignment (Kabsch & Sander, 1983). DSSP has since become a database of secondary structure assignments and other data for all protein entries in the Protein Data Bank (PDB) (Joosten *et al.*, 2011). The DSSP software is available as web service or standalone program, written in C (http://swift.cmbi.ru.nl/gv/dssp/). Despite its popularity and the eminent importance of the concept for structural biology, the algorithm appears not to have been ported to other programming languages.
In the context of our Java Program Collection for Structural Biology and Biophysical Chemistry (Hofmann & Wlodawer, 2002), and specifically for the application SBAL (Wang *et al.*, 2012), a tool for structure-based sequence alignments, we have implemented the DSSP algorithm in Java. To avoid naming confusion with the original DSSP, the Java application is called ASSP.

3. Description of working concept

At the current stage, ASSP works in a similar fashion as DSSP. The user can read a three-dimensional protein structure in PDB format and the software will analyse all geometric and secondary structure (H-bonding) parameters as DSSP. Parsing of the PDB file includes a check for hydrogen atoms being present. If so, the provided hydrogen atoms are kept and used for analysis. If no hydrogen atoms are present in the PDB file, the amide hydrogen atoms will be modelled.
Results are reported either to the terminal or can be saved into an ASCII file. The ASSP API also allows for integration with other Java software.
ASSP has a few methods aiming at particular usage for modelling (e.g. structure-based amino acid sequence alignments), and we plan to implement further model analysis tools in the future.

3.1 Output formats

The user can choose to have the results output in DSSP format (we have used the same format as that delivered by the DSSP web service at http://swift.cmbi.ru.nl/gv/dssp/) or in ASSP format. Apart from a more generous use of spaces between individual parameters, ASSP reports all residues by their original name as listed in the PDB file. For comparison, DSSP addresses residues by a sequential index.

3.2 Comparison of ASSP and DSSP results

The statistics delivered in the header of the original DSSP output by Kabsch and Sander are not currently included in our implementation.
For benchmarking results, please see Java applications for secondary structure analysis of proteins.

4. Operation

4.1 From the terminal

The program can be started from the terminal by

```
java -jar assp_v0.9_java1.6.jar [options]
```

The available options are as follows:

Option	Explanation	Default value
-debug	Debug option.<br>More verbose output; the temporary PDB file `tmp.pdb` is kept in the working directory.	-
-assp	Generate ASSP-style output.	-
-dssp	Generate DSSP-style output.	-
-fasta	Generate FASTA-formatted sequence.	-
-h	Print help.	-
-horiz	Generate secondary structure/sequence alignment similar to PSIPRED's HFORMAT.	-
-i {file name}	Input structure in PDB file format.	-
-noa	No calculation of accessible surface.	
-o {file name}	Save results in this file.	-

4.2 Examples

Analyse secondary structure and show results in ASSP-formatted output on the terminal:

```
java -jar assp_v0.9_java1.6.jar 1kp5.pdb
```

Analyse secondary structure and save results in ASSP-formatted output:

```
java -jar assp_v0.9_java1.6.jar -i 1kp5.pdb -assp -o 1kp5.assp
```

Analyse secondary structure and save results in DSSP-formatted output:

```
java -jar assp_v0.9_java1.6.jar -i 1kp5.pdb -dssp -o 1kp5.dssp
```

Extract FASTA-formatted sequences from a PDB file and save result in a new file:

```
java -jar assp_v0.9_java1.6.jar -i 1kp5.pdb -fasta -o 1kp5.fa
```

5. Explanation of output formats

5.1 ASSP format

```
   .-- sequential residue number, including chain breaks as extra residues
   |     .-- original PDB residue number, not necessarily sequential, may contain letters
   |     |   .-- chain identifier
   |     |   | .-- amino acid sequence in one letter code
   |     |   | |.-- cysteine bridge identifier
   |     |   | || .-- secondary structure summary based on columns 21-43
   |     |   | || | .-- 3-turns/helix
   |     |   | || | |.-- 4-turns/helix
   |     |   | || | ||.-- 5-turns/helix
   |     |   | || | |||.-- geometrical bend
   |     |   | || | ||||.-- chirality
   |     |   | || | |||||.-- beta bridge label
   |     |   | || | ||||||.-- beta bridge label
   |     |   | || | |||||||  .-- beta bridge partner chain ID and residue number
   |     |   | || | |||||||  |      .-- beta bridge partner chain ID and residue number
   |     |   | || | |||||||  |      |       .-- beta sheet label
   |     |   | || | |||||||  |      |       |   .-- solvent accessibility
   |     |   | || | |||||||  |      |       |   |       .-- hydrogen bonds (acceptor residue number, E in kJ/mol)
   |     |   | || | |||||||  |      |       |   |       |                                                 .-- cosine of angle C=O (i),(i-1)
   |     |   | || | |||||||  |      |       |   |       |                                                 |     .-- bend angle Calpha (i-2),(i),(i+2)
   |     |   | || | |||||||  |      |       |   |       |                                                 |     |      .-- dihedral angle Calpha (i-1),(i),(i+1),(i+2)
   |     |   | || | |||||||  |      |       |   |       |                                                 |     |      |       .-- dihedral angle C(i-1)-N(i)-Calpha(i)-C(i)
   |     |   | || | |||||||  |      |       |   |       |                                                 |     |      |       |      .-- dihedral angle N(i)-Calpha(i)-C(i)-N(i+1)
   |     |   | || | |||||||  |      |       |   |       |                                                 |     |      |       |      |       .-- Calpha coordinates
   |     |   | || | |||||||  |      |       |   |       |                                                 |     |      |       |      |       |
   |     |   | || | |||||||  |      |       |   |       ----------------------------------------------    |     |      |       |      |       -----------------------
   |     |   | || | |||||||  |      |       |   |                                                  1         1         1         1         1         1         1         1
....;....1....;....2....;....3....;....4....;....5....;....6....;....7....;....8....;....9....;....0....;....1....;....2....;....3....;....4....;....5....;....6....;....7
         0         0         0         0         0         0         0         0         0         0         0         0         0         0         0         0         0
#     RESIDUE  AA STRUCTURE  BP1     BP2      ACC       N-H-->O      O-->H-N      N-H-->O      O-->H-N    TCO   KAPPA  ALPHA   PHI    PSI     X-CA     Y-CA     Z-CA
   1     1   A ca                               9                A  24 ,-0.1               A  23 ,-0.1                               -21.8    4.288    0.664    -5.547
   2     2   A G       +                      131                A   4 ,-0.2               A  23 ,-0.1   0.841         117.3   65.5   30.9    2.439   -0.322    -8.693
  23    23   A P  E     -Ab  A  44   A 129  A  61                A 129 ,-2.6               A  25 ,-0.4  -0.422   27.6 -150.1  -68.0  135.8    7.387   67.928    -0.110
  24    24   A I  E     -Ab  A  43   A 130  A   4                A  43 ,-1.7               A  26 ,-0.4  -0.898   13.3 -171.4 -112.6  139.8    6.074   65.618     2.618
  25    25   A L  E     -Ab  A  42   A 131  A   1   A  23 ,-0.4  A 131 ,-2.4               A  27 ,-0.4  -0.995    5.5 -169.4 -128.7  125.5    2.604   64.117     3.015
```

5.2 DSSP format

```
    .-- sequential residue number, including chain breaks as extra residues
    |    .-- original PDB residue number, not necessarily sequential, may contain letters
    |    | .-- chain identifier
    |    | | .-- amino acid sequence in one letter code
    |    | | |.-- cysteine bridge identifier
    |    | | || .-- secondary structure summary based on columns 19-34
    |    | | || | .-- 3-turns/helix
    |    | | || | |.-- 4-turns/helix
    |    | | || | ||.-- 5-turns/helix
    |    | | || | |||.-- geometrical bend
    |    | | || | ||||.-- chirality
    |    | | || | |||||.-- beta bridge label
    |    | | || | ||||||.-- beta bridge label
    |    | | || | |||||||   .-- beta bridge partner resnum
    |    | | || | |||||||   |   .-- beta bridge partner resnum
    |    | | || | |||||||   |   |.-- beta sheet label
    |    | | || | |||||||   |   ||   .-- solvent accessibility
    |    | | || | |||||||   |   ||   |     .-- hydrogen bonds (acceptor sequential residue number, E in kJ/mol)
    |    | | || | |||||||   |   ||   |     |                                           .-- cosine of angle C=O (i),(i-1)
    |    | | || | |||||||   |   ||   |     |                                           |    .-- bend angle Calpha (i-2),(i),(i+2)
    |    | | || | |||||||   |   ||   |     |                                           |    |     .-- dihedral angle Calpha (i-1),(i),(i+1),(i+2)
    |    | | || | |||||||   |   ||   |     |                                           |    |     |      .-- dihedral angle C(i-1)-N(i)-Calpha(i)-C(i)
    |    | | || | |||||||   |   ||   |     |                                           |    |     |      |     .-- dihedral angle N(i)-Calpha(i)-C(i)-N(i+1)
    |    | | || | |||||||   |   ||   |     |                                           |    |     |      |     |      .-- Calpha coordinates
    |    | | || | |||||||   |   ||   |     |                                           |    |     |      |     |      |
    |    | | || | |||||||   |   ||   |     ----------------------------------------    |    |     |      |     |      ------------------
    |    | | || | |||||||   |   ||   |                                                 |    |      1         1         1         1         1         1         1         1
....;....1....;....2....;....3....;....4....;....5....;....6....;....7....;....8....;....9....;....0....;....1....;....2....;....3....;....4....;....5....;....6....;....7
         0         0         0         0         0         0         0         0         0         0         0         0         0         0         0         0         0
  #  RESIDUE AA STRUCTURE BP1 BP2  ACC     N-H-->O    O-->H-N    N-H-->O    O-->H-N    TCO  KAPPA ALPHA  PHI   PSI    X-CA   Y-CA   Z-CA
    1    1 A T              0   0   57      0, 0.0     4,-0.1     0, 0.0   204,-0.1   0.000 360.0 360.0 360.0-150.6   28.5   47.4   15.6
    2    2 A G        +     0   0   68    241,-0.2     3,-0.1     2,-0.1   159,-0.1   0.202 360.0   6.1 159.8 -13.4   26.9   47.1   19.0
```

5.3 Geometric parameters

<u>Structure summary</u>

H	α-helix
B	Residue in isolated β-bridge
E	Extended strand, participating in β-ladder
G	3_{10} helix
I	π-helix
T	H-bonded turn
S	Bend

<u>n-Turns</u>

>	Start
<	End
X	Coincidence of '>' and '<'
3,4,5	Residues bracketed by n-turn

<u>Bend</u>

S	Curvature at the central residue of five residues is larger than 70.0°

<u>Chirality</u>

+,-	Sign of the dihedral angle between four consecutive residues (i-1),i,(i+1),(i+2)

<u>Letters</u>

β-strand interface.

<u>BP1, BP2</u>

β-bridge partners.

<u>Letter</u>

β-sheet label.

<u>Accessible surface (ACC)</u>

Number of water molecules in contact with this residue×10, or residue water exposed surface in Å^2.

<u>Hydrogen bonds</u>

An entry under 'N-H-->O' of residue i means that N-H of i is H-bonded to C=O of the listed residue. The electrostatic H-bond energy is given in kcal/mol.
There are two columns for each type of H-bond, to allow for bifurcated H-bonds.

<u>TCO</u>

Cosine of angle between C=O of residue i and C=O of residue (i-1). For alpha-helices, TCO is near +1, for beta-sheets TCO is near -1. Not used for structure definition.

<u>Kappa</u>

Virtual bond angle (bend angle) defined by the three Cα atoms of residues (i-2),i,(i+2). Used to define bend (structure code 'S').

Alpha

Virtual torsion angle (dihedral angle) defined by the four Cα atoms of residues (i-1),i (i+1),(i+2). Used to define chirality (structure code '+' or '-').

Phi

IUPAC peptide backbone torsion angle, C(i-1)-N(i)-Cα(i)-C(i)

Psi

IUPAC peptide backbone torsion angle, N(i)-Cα(i)-C(i)-N(i+1)

X-CA, Y-CA, Z-CA

Cα coordinates of this residue.

6. References

Hofmann A. & Wlodawer A. (2002) PCSB - a program collection for structural biology and biophysical chemistry. *Bioinformatics* **18**, 209-210.

Joosten R.P., te Beek T.A.H., Krieger E., Hekkelman M.L., Hooft R.W.W., Schneider R., Sander C. & Vriend G. (2011) A series of PDB related databases for everyday needs. *Nucleic Acids Res* **39**, D411-9.

Kabsch W. & Sander C. (1983) Dictionary of protein secondary structure: pattern recognition of hydrogen-bonded and geometrical features. *Biopolymers* **22**, 2577-2637.

Wang C.K., Broder U., Weeratunga S.K., Gasser R.B., Loukas A. & Hofmann A. (2012) SBAL: a practical tool to generate and edit structure-based amino acid sequence alignments. *Bioinformatics* **28**, 1026-1027.

7. Appendix: Copyright, Licence and Disclaimer

COPYRIGHT

Copyright (c) by
Hofmann Laboratory, Structural Chemistry Program
Eskitis Institute, Griffith University.
http://www.structuralchemistry.org/pcsb/
All rights reserved.

LICENCE

The authors grant you a non-exclusive, royalty-free licence to use this software, provided that

i) the software is only used for not-for-profit applications;
ii) you do not decompile, reverse engineer or modify the object code;
iii) you do not utilise the software in a manner which is disparaging to the authors;
iv) usage of this software is properly cited.

DISCLAIMER

This software is provided "as is", without a warranty of any kind. All express or implied conditions, representations and warranties, including any implied warranty of merchantability, fitness for a particular purpose or non-infringement, are hereby excluded. The authors shall not be liable for any damages suffered by the user as a result of using or distributing the software or its derivatives. In no event will the authors be liable for any lost revenue, profit or data, or for direct, indirect, special, consequential, incidental or punitive damages, however caused and regardless of the theory of liability, arising out of the use of or inability to use software, even if the authors have been advised of the possibility of such damages.
