# Supplementary material for: An integrated Java tool for generating amino acid sequence alignments with mapped secondary structure elements
Source: 3 Biotech. 2014 May 20;5(1):87–92. doi: 10.1007/s13205-014-0222-0 (PMC4327748; doi:10.1007/s13205-014-0222-0)
Supplement: Supplementary file 1 — Supplementary material 1 (ZIP 15968 kb) [file 13205_2014_222_MOESM1_ESM.zip › 3biotech/sbal_v1.5_manual.pdf]

SBAL v1.5

Table of Contents

1 Introduction........2
2 System requirements and program limitations........2
3 Program description........3
3.1 Getting started........3
3.2 Improving alignment by addressing single sequences........3
3.3 Improving alignment by addressing multiple sequences........4
3.4 Miscellaneous........4
4 Menu description........4
4.1 File........4
4.2 Edit........5
4.3 Tools........6
4.4 Analysis........8
4.5 Help........9
5 References........9
6 Appendix: Licence and Disclaimer........10

1 Introduction

SBAL stands for Structure Based Amino Acid Sequence Alignment and is intended for multiple protein sequence alignments guided by secondary structure elements. The program provides automatic and semi-automatic alignment features, and also possesses manual editing capabilities.
Using sequences provided either as individual PSIPRED (Bryson *et al.*, 2005) output files, FASTA files, DSSP (Kabsch & Sander, 1983) output files or PDB files, SBAL calculates a multiple sequence alignment using a position-specific global alignment algorithm that accounts both for structural as well as for sequence homology.
Where sequences are provided in FASTA format, existing secondary structure information will be searched for in the working directory (PSIPRED files). If no PSIPRED files can be found, secondary structure will be predicted by an in-built 3-layer neural network. Using DSSP output, amino acid sequences from proteins with known three-dimensional structures can be included in the alignments. Multiple sequence alignments in FASTA, MSF and ClustalW format can also be loaded.

For datasets with large numbers of sequences, SBAL will analyse distances from initial pairwise alignments and perform a K-means clustering before computing position-specific alignments. After all clustered sequences have been dealt with, the individual alignments will be aligned. The user can specify when this option is to be chosen by changing the minimum number of sequences required for K-means clustering (see ***Preferences***).

Visual representation of the alignment uses green and red background colour to map helical and beta-strand secondary structure onto individual sequences. Cysteine residues are coloured yellow. The colour highlighting can be turned off by the user.

The editorial features of SBAL allow the user to modify the alignment in a semi-automated fashion. Individual sequences can be moved within the alignment, the position of amino acids in respect to the other sequences can be changed by introducing gaps, and sequences can be added/removed at any time. N- and C-terminal peptides can be excluded from the alignment. Alignments are saved in three file types: an SBAL-specific format (`*.sba`), FASTA format (`*.fa`), and HTML (`*.htm`) which allows a convenient visualisation of the alignment in web browsers or suitable word processing software, as well as upload of alignments to the web for dissemination.

SBAL is part of the PCSB (Hofmann & Wlodawer, 2002) project in our laboratory. When using SBAL, please cite (Wang *et al.*, 2012).

2 System requirements and program limitations

We strongly recommend to use Sun Java (http://www.oracle.com/technetwork/java/index.html).
SBAL has been successfully tested with the following Sun Java versions:

- 1.6.0_03-b05
- 1.6.0_04

The maximum number of sequences that can be loaded into SBAL is 500.

3 Program description

3.1 Getting started

To create an alignment, *de novo* ***Auto-alignment using PSIPRED output*** is selected from the ***Tools*** menu. The user needs to specify the folder where the input files are located, and the program will read sequence and secondary structure information from the provided `*.ss2` or `*.horiz` files. If the directory also contains sequence information as individual FASTA (`*.fa`) files, the sequence names will be resourced from those. SBAL will start the auto-alignment procedures without further user input.

Alternatively, an existing alignment can be loaded either in the SBAL, FASTA, MSF or ClustalW format by clicking on ***Open Alignment*** under the ***File*** menu. Sequences also be added one by one using the ***Add sequence*** or ***Add structure*** feature from the ***Tools*** menu.

The ***Alignment Editor*** pane is separated into two sections whose interface boundary can be adjusted. On the left (ID panel), the names of the individual sequences are displayed, and on the right (sequence panel), the actual sequences are shown in a scrollable window. The progress bar at the very left is active when auto-alignment calculations are being performed.

The secondary structure elements are mapped onto amino acid sequences by colour code (green = α-helix, red = β-strand), which allows for a convenient visual inspection and manual adjustment of the alignment using different functions described in the next section. Cysteine residues are highlighted in yellow. The colour highlighting may be turned off by selecting a range of sequences in the ID panel with `CTRL + LEFT MOUSE BUTTON`.

The cursor functions `LEFT` and `RIGHT` move the cursor within an individual sequence without modifying the alignment. The cursor functions `UP` and `DOWN` move the cursor from one sequence to another. The current selected sequence is highlighted in grey in the ID panel.

The residue number of an individual amino acid in the alignment can be queried, by positioning the mouse on the residue of interest and performing a `CTRL + LEFT MOUSE BUTTON` click.

3.2 Improving alignment by addressing single sequences

The `NUM LOCK` function on the keyboard needs to be off. Using the numeric keypad, single sequences can be moved within the alignment (up/down: `NUMPAD8`/`NUMPAD2`) or with respect to the other sequences (left/right: `NUMPAD4`/`NUMPAD6`). A single sequence can also be moved to the very top (`CTRL + NUMPAD8`) or the very bottom (`CTRL + NUMPAD2`).

Gaps can be introduced into a sequence either by pressing the `- KEY` or the `SPACE BAR`. To delete gaps, use the `BACKSPACE KEY`.

The program automatically deletes gap columns after each modification.

In order to improve the alignment of a single sequence, a set of target sequences can be chosen to which the desired sequence is aligned based on sequence homology. See ***Position-specific alignment*** (4.3.6) below.

3.3 Improving alignment by addressing multiple sequences

Several functions such can also be executed for multiple sequences simultaneously. The simultaneously executed functions are:

- moving sequences within the alignment
- introducing gaps
- deleting gaps
- N- and C-terminal truncation

There are two ways to select multiple sequences for simultaneous activities. In order to make a continuous selection of sequences, `LEFT CLICK` and drag across the names in the ID panel with the mouse. The selection can be released by a `LEFT CLICK` somewhere in the ID panel. The other way is to select single sequences by a `LEFT CLICK` on individual names in the ID panel. A second `LEFT CLICK` on the same sequence releases the selection. After the selection process, click into any of the selected amino acid sequences (text colour blue) in the sequence panel.

3.4 Miscellaneous

Highlighting the secondary structure elements in the entire alignment might use a lot of resources and slow down the response time of the program in large alignments. In order to increase the speed the marking can be removed by `CTRL + LEFT MOUSE BUTTON` on the individual sequence names on the left. To restore the highlighting, follow the same procedure.

Residual N- or C-terminal sequence fragments can be removed by applying ***N- or C-terminal truncation*** found under the menu ***Edit***. The fragments will disappear from the ***Alignment Editor*** and be ignored for all further alignment modifications. When saving the alignment, the presence of a truncation will be indicated by "..." at the N- or C-terminal end in the HTML output file. N-terminally truncation peptides will be included in the amino acid sequence numbering. Truncated peptides are listed for each sequence below the alignment in the HTML output. Truncated peptides are not included in the FASTA-formatted alignment.

Functions to delete or add new sequences to the alignment can be found under the menu ***Edit*** (4.2).

4 Menu description

4.1 File

4.1.1 Open alignment

Reads an existing alignment in SBAL (`*.sba` file), FASTA, MSF or ClustalW format. Only alignments provided in SBAL format include secondary structure information. For all other formats, the program will try to find PSIPRED (`*.ss2` or *.horiz files) and FASTA (`*.fa`) files in the same directory; the secondary structure information will be read from these PSIPRED files. If this process is unsuccessful, secondary structure will be predicted using the built-in single sequence secondary structure prediction.

4.1.2 Save alignment

Saves the current alignment and creates three different output files (`*.htm`, `*.fa`, `*.sba`). The output directory as well as the root file name has to be specified by the user.
The HTML output file allows a convenient visualization of the alignment using any web browser (green = α-helix, red = β-strand, cysteine residues are depicted in bold). The number printed after each line indicates the current amino acid position of the respective sequence. The SBAL output file

is the most convenient way to continue working on an existing alignment with the program.

4.1.3 Divide alignment
With this function, the current alignment can be split into two: the first one ranging from the first sequence to the one that currently harbours the cursor, and the second alignment ranging from the next sequence to the last sequence. The user will be prompted to enter an output directory as well as a root name for the two alignments. SBAL will append “_1” and “_2”, respectively. Three file formats (SBAL, FASTA and HTML) will be written for each of the two alignments.

4.1.4 Exit
Ends the program.

4.2 Edit
4.2.1 Undo
This function restores the alignment into the stage before the most recent operation actioned by the user.

4.2.2 Rename sequence
The names of activated sequences can be changed using this function.

4.2.3 Remove gap columns
Removes any gap columns which may be present in the alignment. This function may only be needed if non-SBAL alignments are loaded.

4.2.4 Move left of gap
The amino acids placed to right of the current cursor position will be moved to the right side of the next gap upstream. This function can also be actioned with the `l key`.

4.2.5 Move right of gap
The amino acids placed to left of the current cursor position will be moved to the left side of the next gap downstream. This function can also be actioned with the `r key`.

4.2.6 N-terminal truncation
This function allows the removal of N-terminal amino acids. The peptide from the very N-terminal amino acid up to the current cursor position will be removed. The program highlights the selected sequence and asks for confirmation to remove the peptide.

4.2.7 C-terminal truncation
This function allows the removal of C-terminal amino acids. The peptide from the current cursor position to the very C-terminal amino acid will be removed. The program highlights the selected sequence and asks for confirmation to remove the peptide.

4.2.8 Add annotation
This will add a new line above the current line (identified by the line cursor in the sequence panel of the ***Alignment Editor***) to be used for free text annotation. The title “Annotation” will be displayed in the ID panel. This can be changed by following the procedure for ***Rename sequence*** (4.2.2).

4.2.9 Preferences
User preferences can be set for the following:

- ***Automatic score calculation*** (default: off). If on, SBAL re-calculates the alignment scores shown in the status bar after each operation actioned by the user.
- ***Helix colour*** can be chosen with the adjacent colour button (default: green).
- ***Strand colour*** can be chosen with the adjacent colour button (default: red).
- ***Cys colour*** can be chosen with the adjacent colour button (default: yellow).
- ***Show secondary structure markup*** enables turning on/off of the background colouring in SBAL.
- ***Cysteine markup in HTML output*** allows to choose between coloured background (***Colour***) defined in ***Cys colour*** or bold type face (***Bold***).
- ***Residues per line in HTML output*** defines the number of amino acid positions within one line in the HTML output.

4.3 Tools
4.3.1 Add sequence
Adds a new sequence to the existing alignment. The selected file must be in one of the following formats:

- PSIPRED `VFormat` (`*.ss2`) or `HFormat` (`*.horiz`)
- FASTA
- DSSP output
- PDB

By default, the new sequence is auto-aligned. This function can be disabled by un-ticking the checkbox ***automatically align sequence***. In that case, the sequence will be added at the bottom of the alignment and C-terminally aligned. The program will also perform a check for any existing identical sequences in the alignment and present the user with the option of removing these. The newly added sequence is shown in magenta.
For sequences provided in FASTA format, SBAL will attempt to obtain secondary structure information from PSIPRED files with the same name in the current directory; if this fails, secondary structure will automatically be predicted.
For PDB files, the user can choose between secondary structure being determined by the implemented ASSP algorithm or based on information in the PDB file (`HELIX` or `SHEET` records, if present).

4.3.2 Delete sequence
The selected sequence will be deleted.

4.3.3 Remove duplicates
The current alignment is checked for duplicate amino acid sequences which can be deleted automatically or by user confirmation.

4.3.4 Add alignment
A preformed alignment can be imported and will be aligned with the current alignment in SBAL.

4.3.5 Auto-alignment
This function calculates an alignment from sequences in individual files. All files to be analysed need to be within in user-specified directory. The files must be in one of the following formats and all have the appropriate extension:

- PSIPRED - file names: `*.ss2` or `*.horiz`
- FASTA - file names: `*.fa`
- DSSP output - file names: `*.dssp`
- PDB - file names: `*.pdb`

When reading a PSIPRED file, SBAL attempts to find a FASTA file with the same name in the current directory; from the FASTA headers, the title of individual sequences are automatically collected.
For sequences provided in FASTA format, secondary structure will automatically be predicted using the built-in prediction algorithm.
For sequences provided in PDB format, SBAL determines whether secondary structure information is provided in the PDB file (`HELIX` and `SHEET` records). The user can then choose whether this information is to be used, or whether secondary structure is to be determined using the implemented ASSP algorithm.

4.3.6 Position-specific alignment

This function allows alignment of the currently active sequence (highlighted by a grey background in the ID panel) to a selected group of sequences which act as a template for position-specific alignment. Template sequences are selected by left-clicking on individual sequences or by dragging the mouse to select a continuous group of sequences in the ID panel. Selected sequences will be highlighted in blue. The user then needs to place the cursor anywhere in the target sequence (gray highlight), and execute the function ***Position-specific alignment***.

4.3.7 Thread

With this function, an amino acid is aligned to a single other sequence. The user needs to select at least two sequences. The sequence ranking highest in the list is used as reference sequence. All other selected sequences below will be aligned to the reference sequence and automatically be re-positioned so they appear just below the reference sequence in the updated alignment. This feature may be useful when adding new sequences to an existing alignment.

4.3.8 Read secondary structure

For the current alignment, the user can specify a directory where PSIPRED files with secondary structure information for the individual sequences are located. Note that FASTA files (`*.fa`) with individual sequences and their titles are needed as well (since the program needs to map the secondary structure onto the correct sequence in the alignment, identified by name of the individual sequence).

4.3.9 Assign secondary structure

Secondary structure elements can be assigned to individual residues and peptides by highlighting the region of interest in the sequence panel. Then, ***Assign secondary structure*** from the Tools menu needs to be selected, which will allow the user to assign one of the three secondary elements (Helix, Strand or Coil).

4.3.10 Alignment preferences

The number of cycles to be performed in automatic PS alignment (***Auto-alignment***) and manual PS alignment (***Position-specific alignment***) can be changed here. The default setting is 1 cycle for either ***Auto-alignment*** or ***Position-specific alignment***.

The number of sequences required for SBAL to switch to K-means clustering can be set under this item, too; the default is 20.

4.4 Analysis

4.4.1 Alignment score

Displays the alignment score for selected sequences in the status bar at the bottom with respect to primary and secondary structure. If no selection is made, the alignment score for the entire alignment is shown.

4.4.2 Pairwise sequence identity

Displays the amino acid sequence identity of two selected sequences within the alignment in the status bar. The first score refers to the comparison of both sequences over the entire length of the alignment:

> *AAIdentity* = Σ positions matched / Σ positions compared
> Only full matches are counted; gaps are included as follows: gap on gap is considered a match, gap on amino acid is considered a mis-match.

The score given in brackets refers to the comparison of both sequences only in areas where amino acids are aligned (“no gaps”):

> *AAIdentityNoGaps* = Σ positions matched / Σ positions compared
> Only full matches are counted; gaps are treated as follows: gap on gap is ignored, gap on amino acid is considered a mis-match.

4.4.3 Distance matrix

This option will calculate a distance matrix for the entire alignment with is displayed in a separate window. The following distances can be chosen: ***AA identity***, ***p distance 1*** and ***p distance 2***.
For ***AA identity***, the distances are calculated using the *AAIdentityNoGaps* parameter defined in section 4.4.2 .
For ***p distance 1***, the p distance is calculated as used in the EMBOSS distmat program (Rice *et al.*, 2000):

> *pDistance1* = 1 – [Σ positions matched / (Σ positions compared + Σ gaps * *gapPenaltyP*)]
> Currently, *gapPenaltyP* = 0.

For ***p distance 2,*** the p distance is calculated as used by the program MEGA (Kumar *et al.*, 2001):

> ***pDistance2*** = Σ positions mis-matched / Σ positions compared
> Gaps are ignored.

4.4.4 Calculate profile

A profile based on the currently selected sequences in the ID panel will be calculated. The profile will be appended as two lines at the bottom of the alignment. One line shows the amino acid sequence of the profile with mapped secondary structure, the other line shows the weighting of each position (calculated as frequency of the particular amino acid and ranked from 0-9). The profile lines can be removed by `CTRL + LEFT MOUSE BUTTON` on either line in the ID panel.

4.4.5 Peptide information

This feature provides an assessment of the physical parameters of a peptide sequence, including molecular mass, estimated molar absorption coefficient, etc. The sequence harbouring the cursor (grey highlight) is submitted to the implemented ***Peptides*** (Hofmann & Wlodawer, 2002) algorithm, and the results are displayed in a popup window.

4.5 Help

4.5.1 License

Shows the license conditions.

4.5.2 About

Displays the program version.

5 References

Bryson K., McGuffin L.J., Marsden R.L., Ward J.J., Sodhi J.S. & Jones D.T. (2005) Protein structure prediction servers at University College London. *Nucl Acids Res* **33**, W36-W38.

Hofmann A. & Wlodawer A. (2002) PCSB - a program collection for structural biology and biophysical chemistry. *Bioinformatics* **18**, 209-210.

Kabsch W. & Sander C. (1983) Dictionary of protein secondary structure: pattern recognition of hydrogen-bonded and geometrical features. *Biopolymers* **22**, 2577-2637.

Kumar S., Tamura K., Jakobsen I.B. & Nei M. (2001) MEGA2: molecular evolutionary genetics analysis software. *Bioinformatics* **17**, 1244-1245.

Rice P., Longden I. & Bleasby A. (2000) EMBOSS: The European Molecular Biology Open Software Suite. *Trends Gen* **16**, 276-277.

Wang C.K., Broder U., Weeratunga S.K., Gasser R.B., Loukas A. & Hofmann A. (2012) SBAL: a practical tool to generate and edit structure-based amino acid sequence alignments. *Bioinformatics* **28**, 1026-1027.

6 Appendix: Licence and Disclaimer

COPYRIGHT

Copyright (c) by
Hofmann Laboratory, Structural Chemistry Program
Eskitis Institute, Griffith University.
http://www.structuralchemistry.org/pcsb/
All rights reserved.

LICENCE

The authors grant you a non-exclusive, royalty-free licence to use this software, provided that

i) the software is only used for not-for-profit applications;
ii) you do not decompile, reverse engineer or modify the object code;
iii) you do not utilise the software in a manner which is disparaging to the authors;
iv) usage of this software is properly cited.

DISCLAIMER

This software is provided "as is", without a warranty of any kind. All express or implied conditions, representations and warranties, including any implied warranty of merchantability, fitness for a particular purpose or non-infringement, are hereby excluded. The authors shall not be liable for any damages suffered by the user as a result of using or distributing the software or its derivatives. In no event will the authors be liable for any lost revenue, profit or data, or for direct, indirect, special, consequential, incidental or punitive damages, however caused and regardless of the theory of liability, arising out of the use of or inability to use software, even if the authors have been advised of the possibility of such damages.
